# The difference and relationship of CD4+ and CD8+ tumour infiltrating lymphocytes in lung adenocarcinoma

**DOI:** 10.18632/oncotarget.26666

**Published:** 2019-02-12

**Authors:** Chaoting Zhang, Zheming Lu, Hongying Huang

**Affiliations:** Hongying Huang: Department of Pathology, New York University Langone Medical Center, New York, NY, USA

**Keywords:** TILs, lung cancer

Lung cancer is the leading cause of cancer-related morbidity and mortality worldwide and non-small-cell lung cancer (NSCLC) represents approximately 85% of all lung cancer patients [1]. Immunotherapies have demonstrated promising clinical efficacy in treatments to patients with NSCLC [2]. In spite of remarkable results, only a fraction of patients respond and thus there is a strong need to explore factors influencing immunotherapy efficacy [2]. Since tumour infiltrating T cells (TILs) play a critical role in immunotherapies, it is essential to comprehensively profiled subtype, frequency, phenotype and spatial distribution of TILs, which could affect NSCLC response to immunotherapy [3].

To evaluate subtype, frequency, phenotype and spatial distribution of TILs in patients with NSCLC, we isolated CD4+ and CD8+ TILs in tumor centers and margins by flow cytometry on 80 tumor samples from 20 patients [4]. We found that the amount of CD4+ TILs was significantly higher than CD8+ TILs. In addition, CD4/CD8 T-cell ratios in different regions of tumour center were more similar than those in different regions of tumour margin, although CD4/CD8 T-cell ratios hugely varied in different regions of same tumour. Since T-cell receptor (TCR) expressed by TILs is the key to specifically identify tumor antigens, it is essential to assess TCR repertoire of CD4+ and CD8+ TILs in different regions. Therefore, we performed highthroughput TCR sequencing on 27 and 25 samples of CD4+ and CD8+ TILs from seven patients. Our results showed that TCR repertoire clonality of CD8+ TILs was significantly higher than that of CD4+ TILs. In addition, we found that CD8+ TIL repertoire across center regions was more similar than that across margin regions although TCR repertoire of CD4+ and CD8+ TILs significantly varied in center and margin of all tumors. Moreover, the amount and TCR repertoire of CD4+ and CD8+ TILs in tumor centers were associated with a patient’s risk of relapse.

In light of these findings, we could anticipate that heterogeneity in the subtype, amount, and TCR repertoire of TILs in different tumour regions could be due to spatial difference in nature and abundance of tumour antigens within the same tumor [5, 6]. Furthermore, TILs in tumour centers were more similar than those in tumour margins, and moreover the amount and TCR repertoire of CD4+ and CD8+ TILs in tumor centers were significantly associated with progression-free survival, which suggested that TILs in tumour centers were more likely to be tumour-specific. However, we can’t exclude the possibility that characteristics of TILs in different regions could be due to heterogeneity of tumor architecture, potentially within the tumor vasculature and lymphatics which could affect traffic and infiltration of T cells.

These data were critical for immunotherapies for NSCLC. First, multiple biopsies were needed to obtain a comprehensive overview of TILs to an individual NSCLC. Second, compared with CD8+ TILs, the amount and TCR diversity of CD4+ TILs are generally higher. Third, adoptive cell therapy using TILs in tumour center could be more likely to achieve clinical efficacy in patients with NSCLC. Last, characteristics of TILs in tumour centers could more accurately predict response to immunotherapies, such as anti-PD1 immunotherapy.

In summary, our continuous work would further profile characteristics of TILs in tumour center and identify tumour-specific TILs which could be applied to immunotherapy for patients with NSCLC.
